# Reducing meat consumption in the USA: a nationally representative survey of attitudes and behaviours

**DOI:** 10.1017/S1368980017004190

**Published:** 2018-03-26

**Authors:** Roni A Neff, Danielle Edwards, Anne Palmer, Rebecca Ramsing, Allison Righter, Julia Wolfson

**Affiliations:** 1 Department of Environmental Health & Engineering, Johns Hopkins Bloomberg School of Public Health, 615 N. Wolfe Street, Baltimore, MD 21205, USA; 2 Department of Health Policy & Management, Johns Hopkins Bloomberg School of Public Health, Baltimore, MD, USA; 3 Center for a Livable Future, Johns Hopkins Bloomberg School of Public Health, Baltimore, MD, USA; 4 Department of Health, Behavior, & Society, Johns Hopkins Bloomberg School of Public Health, Baltimore, MD, USA; 5 School of Culinary Science & Nutrition, The Culinary Institute of America, Hyde Park, NY, USA; 6 Department of Health Management and Policy, University of Michigan School of Public Health, Ann Arbor, MI, USA

**Keywords:** Meat consumption, Dietary behaviours, Meat reduction, Dietary attitudes

## Abstract

**Objective:**

Excess meat consumption, particularly of red and processed meats, is associated with nutritional and environmental health harms. While only a small portion of the population is vegetarian, surveys suggest many Americans may be reducing their meat consumption. To inform education campaigns, more information is needed about attitudes, perceptions, behaviours and foods eaten in meatless meals.

**Design:**

A web-based survey administered in April 2015 assessed meat reduction behaviours, attitudes, what respondents ate in meatless meals and sociodemographic characteristics.

**Setting:**

Nationally representative, web-based survey in the USA.

**Subjects:**

US adults (*n* 1112) selected from GfK Knowledgeworks’ 50 000-member online panel. Survey weights were used to assure representativeness.

**Results:**

Two-thirds reported reducing meat consumption in at least one category over three years, with reductions of red and processed meat most frequent. The most common reasons for reduction were cost and health; environment and animal welfare lagged. Non-meat reducers commonly agreed with statements suggesting that meat was healthy and ‘belonged’ in the diet. Vegetables were most often consumed ‘always’ in meatless meals, but cheese/dairy was also common. Reported meat reduction was most common among those aged 45–59 years and among those with lower incomes.

**Conclusions:**

The public and environmental health benefits of reducing meat consumption create a need for campaigns to raise awareness and contribute to motivation for change. These findings provide rich information to guide intervention development, both for the USA and other high-income countries that consume meat in high quantities.

The USA has the fifth highest per capita meat consumption in the world^(^ ^1^ ^)^. Meat consumption in the USA exceeds healthy levels by 20–60 % based on recommendations in the 2015–2020 Dietary Guidelines for Americans^(^ ^2^ ^,^ ^3^ ^)^. Excess consumption of meat, especially red and processed meats, is associated with health conditions including heart disease^(^ ^4^ ^–^ ^6^ ^)^, stroke^(^ ^6^ ^,^ ^7^ ^)^, type 2 diabetes^(^ ^6^ ^,^ ^8^ ^)^, obesity^(^ ^9^ ^)^ and some cancers^(^ ^4^ ^,^ ^5^ ^)^. Red and processed meats are associated with higher overall, cardiovascular and cancer mortality rates^(^ ^10^ ^)^. The WHO has determined that red meat in general is ‘probably carcinogenic to humans’ and processed meat is ‘carcinogenic to humans’^(^ ^11^ ^)^.

The environmental impact of meat consumption includes contamination of water, air and soil, and far greater use of resources such as water and fuel compared with other food sources^(^ ^12^ ^,^ ^13^ ^)^. Livestock, particularly cattle, are responsible for 14·5 % of global greenhouse gas emissions^(^ ^14^ ^)^. Non-therapeutic antibiotic use in industrial food animal production leads to the development of antibiotic-resistant bacteria, which threatens the effectiveness of antibiotics in human medicine^(^ ^12^ ^)^.

Many Americans have strong preferences for meat^(^ ^15^ ^)^. Yet, in surveys, many report that they have reduced their meat consumption or aspire to do so^(^ ^16^ ^–^ ^20^ ^)^. For example, 39 and 32 % of Americans, respectively, said they ate less meat than they did three years earlier in 2012 and 2015 surveys by National Public Radio/Thompson Reuters^(^ ^17^ ^,^ ^18^ ^)^. In a 2014 survey by FGI Research, 16 % of Americans stated they had cut back on meat in the past year^(^ ^19^ ^)^. In all three surveys, about a third of Americans said they hoped to reduce the amount of meat they consumed in the future^(^ ^17^ ^–^ ^19^ ^)^. In a 2015 US survey by the Vegetarian Resource Group and Harris Poll, 36 % of respondents reported eating at least one vegetarian meal per week, 10 % said they ate vegetarian more than half the time and 3·4 % self-identified as never eating meat (vegetarian and/or vegan)^(^ ^21^ ^)^.

Little is known about what people eat when they reduce their meat consumption without going fully vegetarian. It is likely that substitutions vary based on reasons for reduction^(^ ^22^ ^)^, cultural norms and the diverse definitions of ‘meat’. One European study found that 76 % of meat reducers ate fish in meatless meals, 49 % eggs, 34 % cheese, 26 % imitation meats such as vegetarian burgers, 17 % lentils or beans, 14 % tofu and 9 % nuts^(^ ^23^ ^)^. It is unknown how generalizable this information is to the USA. A 2017 study using the US National Health and Nutrition Examination Survey (NHANES) examined diet quality among those reporting that they did not eat food animals on either of two separate days^(^ ^24^ ^)^. About half of that group, which was classified as ‘non-meat eaters’, self-identified as vegetarian. Overall, non-meat eaters had higher Healthy Eating Index scores than meat eaters, but the research identified a wide spread in diet quality among non-meat eaters. Higher diet quality was seen among non-meat eaters who were older, female and higher income, among other categories.

Given that a significant portion of the US population may be purposefully reducing their meat consumption without fully abstaining (becoming vegetarian or vegan), more information is needed about the specific foods they choose instead, because the level of benefit to health or the environment will vary considerably based on the choices^(^ ^25^ ^)^. For example, cheese is generally high in saturated fat, Na and energy, and while its environmental footprint is lower than that of beef, it is higher on average than that of poultry, most fish and most vegetarian imitation meats^(^ ^3^ ^,^ ^26^ ^)^. The above-cited 2017 study found that while healthy plant-based diets conferred considerable advantages in preventing CHD, unhealthy plant-based diets increased the risk^(^ ^25^ ^)^. Recognizing these factors, there is a need for deeper perspectives on how people understand and think about meat reduction.

We conducted a nationally representative survey of US adults to learn about what is eaten in meatless meals, attitudes and perceptions towards meat reduction, and to build upon and add depth to previous research on meat reduction behaviours in the USA and other high meat-consuming countries. Recognizing the popularity of cheese and dairy in the US diet and speculating that it would be common to replace animal products with other animal products, we hypothesized that cheese/dairy would be the most common food that respondents indicated eating in meatless meals. We further hypothesized based on prior polling data that health and cost would be the primary reasons for reducing meat consumption among those continuing to eat some meat.

## Methods

A web-based survey instrument was developed using both new questions and questions adapted from sources including the National Public Radio/Thompson Reuters and FGI Research surveys mentioned above^(^ ^17^ ^,^ ^19^ ^)^. The survey was fielded in April 2015 using the research firm, GfK Knowledgeworks. GfK maintains an online panel of approximately 50 000 members recruited using equal-probability address-based sampling. The sampling frame covers 97 % of US households. To improve representation, GfK provides Internet access and devices to those who lack them, and oversamples census blocks with high percentages of African-American and Hispanic residents. GfK panel members are eligible for modest cash and prize drawings not linked to participation in specific surveys. In addition to pre-survey weighting to reflect annually updated benchmark US population distributions, GfK provides post-survey sample weights based on seven variables to correct for sampling and non-response biases.

The survey was sent to 1568 panel members of whom 1137 completed the survey. We excluded twenty-five who unrealistically completed the survey in under 4 min, leading to a 73 % completion rate and a final sample size of 1112. The full survey instrument included questions about cooking attitudes and behaviours developed for a separate study^(^ ^27^ ^)^. The question text is provided in the online supplementary material, Supplemental File 1. Supplemental File 2 provides further documentation of sampling and other methods using the CHERRIES checklist for reporting results of Internet e-surveys.

### Measures

#### Types of meat

Meat was classified into four mutually exclusive categories: (i) red meat, fresh or frozen (beef, pork, lamb, duck, etc.); (ii) processed meat (bacon, hot dogs, deli meats, sausages, etc.); (iii) poultry, fresh, frozen or canned/bagged (chicken, turkey, etc.); and (iv) seafood, fresh, frozen or canned/bagged (fish, shrimp, crab, clams, etc.) We did not overtly define ‘meat’ as we were interested in responses based on consumers’ own interpretations.

#### Meat reducer status

The primary outcome in multivariate regressions was ‘meat reducer’ *v.* ‘non-reducer’. Meat reducers were defined as individuals reporting ‘a lot less’ or ‘slightly less’ consumption *v.* three years ago for one or more of the four meat types examined. Individuals reporting no change or an increase were called ‘non-reducers’. Using the same criteria, we also identified individuals as either reducers or non-reducers for each of the four meat types (red meat, processed meat, poultry, seafood).

#### Meat consumption

Participants rated their consumption frequency for each of the four meat types in the past 7 d on an 8-point ordinal scale. Three items at the high end of the scale were collapsed to yield a 6-point measure (not at all, 1 time/week, 2–4 times/week, 5–6 times/week, 1 time/d, more than 1 time/d). Frequency of consuming red and processed meat were significant predictors of meat reduction, and were included as covariates in the regression models. Meat reducers were also asked how they had reduced their meat consumption, with five options including ‘eating smaller portions’, ‘cutting meat from certain meals’ and ‘skipping meat one day/per week’.

#### Food consumption in meatless meals

All respondents indicated the frequency with which they replaced meat with specific foods in their non-meat meals: fake meats (‘such as meat-free nuggets, crumbles or strips, veggie burgers or vegetarian sausages’), cheese or other dairy, eggs, nuts, fish or seafood, tofu, seitan or tempeh (herein called ‘tofu’ for short), beans, grains and vegetables. Responses were measured on a 5-point scale from ‘don’t eat’ to ‘always eat’. In the current paper we use the more formal term, ‘imitation meats’, to refer to the fake meat category.

#### Sociodemographic covariates

Demographic covariates used in the regression models included age (18–29, 30–44, 45–59, ≥60 years), household income (≤$US 24 999, $US 25 000–49 999, $US 50 000–74 999, ≥$US 75 000), gender, parent/step-parent living with a child under age 18 years, race/ethnicity (White, non-Hispanic; Black, non-Hispanic; Hispanic; other); and education (less than high school; high school; some college; bachelor’s degree or higher). Race/ethnicity and education were included for conceptual reasons, although these did not generally yield statistically significant results. Additional demographic measures (including four- and nine-region geographic locations) were examined but excluded from the final model because no statistically significant relationships to the outcomes were identified.

#### Attitudes

Meat reducers were asked whether any of the following reasons helped explain their changed consumption: health, animal welfare, environment, cost or other. Non-meat reducers were asked to rank their level of agreement on a 7-point scale with eight statements about eating less meat, including: ‘don’t know how to cook meatless’, ‘don’t like the taste’, ‘too expensive’ and ‘a healthy diet includes meat’.

The instrument was reviewed by content experts and pilot-tested using a random sample of twenty-five participants in the survey firm’s panel.

### Analysis

We performed descriptive analyses of meat consumption frequency and reduction, reasons for meat reduction and non-reduction, and what respondents ate in their non-meat meals. We used tests to assess demographic differences between meat reducers and non-reducers, differences by income in reasons for meat reduction, and differences by parental status, gender and income in non-reducers’ agreement with statements related to meat reduction. We then assessed factors associated with meat reduction using multivariable logistic regression. Separate regressions were performed for the overall ‘meat reducer’ category, and for reducers of red meat, processed meat, poultry and seafood. We considered three variable groups for inclusion: demographics, meat consumption frequency and foods eaten in meatless meals. We did not include attitudes towards meat reduction in the model because reducers and non-reducers were asked different questions. Variables (other than race/ethnicity and education) were excluded if their coefficients did not reach statistical significance at the *α*=0·05 level. The models were checked for specification error and goodness-of-fit was assessed with the Hosmer–Lemeshow test. The presented analyses incorporated GfK’s provided survey weights to present nationally representative results. Statistical analyses were performed using the statistical software package Stata version 13.1, with some descriptive analyses performed in Microsoft^®^ Excel version 14.5 (2011).

## Results

Table 1 describes sample demographics overall and by meat reducers and non-reducers. The weighted sample was similar to the national averages for age, income, gender, education and geography, and oversampled Black and Hispanic respondents. Sixty-six per cent of survey participants described themselves as having reduced their consumption in at least one meat category compared with three years ago. We characterized these individuals for further analyses as ‘meat reducers’ even if they increased or maintained consumption of other meat types.

**Table 1:** Demographic characteristics generally and by ‘meat reducers’ in a nationally representative adult sample: USA, 2015

	Survey (*n* 1112)			National	% Meat reducers	% Non-meat
	*n*	Unweighted %	Weighted %	comparison %	in category	reducers in category
	1112	100·0	100·0	100·0	66	33
Age**						
18–24 years	97	8·7	11·3	11·3	10	13
25–34 years	173	15·6	17·9	16·7	16	23
35–44 years	167	15·0	17·5	16·7	16	21
45–54 years	205	18·4	17·0	19·0	19	12
55–64 years	239	21·5	18·9	17·3	20	17
≥65 years	231	20·8	17·4	18·9	19	14
Household income						
<$US 10 000	44	4·0	5·0	6·8	3	6
$US 10 000–24 999	135	12·1	12·9	16·8	10	14
$US 25 000–49 999	226	20·3	22·5	26·2	22	23
$US 50 000–74 999	210	18·9	18·4	19·2	22	17
≥$US 75 000	497	44·7	41·2	30·9	43	40
Gender*						
Female	567	51·0	51·8	52·4	45	55
Race/ethnicity						
White	792	71·2	65·5	82·4	66	66
Black	106	9·5	11·5	9·9	12	11
Other	87	7·8	7·8	7·7	8	7
Hispanic						
Hispanic	127	11·4	15·2	11·3	15	16
Non-Hispanic	985	88·6	84·8	88·7	85	84
Education						
Less than high school	97	8·7	12·4	13·0	14	12
High school	319	28·7	29·6	30·3	31	29
Some college	319	28·7	28·7	28·7	28	29
Bachelor’s degree or higher	377	33·9	29·2	28·0	27	30
US region						
Northeast	212	19·1	18·2	18·1	18·0	18·8
Midwest	254	22·8	21·4	21·3	20·3	23·3
South	408	36·7	37·1	37·2	37·7	35·8
West	238	21·4	23·4	23·5	23·9	22·1

χ^2^ test comparing percentages of meat reducers and non-reducers: *P<0·05, **P<0·01.

### Meat consumption frequency

Table 2 describes meat consumption and reduction patterns. Respondents most frequently reported consuming red meat and poultry two to four times weekly (42 and 37 %, respectively) and processed meat once weekly (30 %). More than half (52 %) said they had not consumed any seafood in the past week. Sixteen per cent of respondents reported consuming at least one meat type daily, including 9 % who consumed red meat daily. Fifty-five per cent of respondents reporting reducing processed meat and 41 % reduced red meat. Of those who reported reducing red and processed meat, 37 % said they had increased poultry or seafood consumption. Across the sample, 21 % also said they had reduced poultry and 26 % seafood, while 10 % said they had reduced meat consumption in all four categories.

**Table 2:** Frequency of meat consumption in the past week, and reported reduction in the past three years (all respondents), in a nationally representative adult sample: USA, 2015

	Red meat	Processed meat	Poultry	Seafood
	%	%	%	%
Frequency				
Not at all	21	29	27	52
1 time/week	19	30	20	23
2–4 times/week	42	29	37	15
5–6 times/week	8	5	9	3
1 time/d	5	4	4	3
>1 time/d	4	3	3	2
Reduction				
A lot less	20	27	11	14
Slightly less	21	28	10	12
About the same	53	41	58	57
Slightly more	5	4	18	14
A lot more	1	0	4	4

Table 3 presents factors associated with meat reduction in any category and with reducing each of the four meat types. Among demographic predictors assessed, significant relationships were observed between overall, red and processed meat reduction and age. Reductions were most common among those aged 45–59 years compared with those aged 18–29 years (overall: OR=2·04, *P*<0·001; red: OR=1·58, *P*<0·05; processed: OR=1·67, *P*<0·01). Meat reduction overall was also elevated among those aged ≥60 years (OR=1·67, *P*<0·05). Household income was also associated with reducing meat consumption. Respondents with income ≤$US 24 999 were more likely to reduce overall (OR=1·60, *P*=0·05), poultry (OR=2·44, *P*<0·001) and seafood (OR=1·75, *P*<0·01) consumption compared with those with household incomes of ≥$US 75 000. Men were less likely than women (OR=0·77, *P*<0·05) to reduce overall meat consumption, but gender was not a significant predictor of reducing any of the specific meat types. Parents of children under age 18 years were less likely to reduce their overall (OR=0·68, *P*<0·05), red (OR=0·63, *P*=0·01) and poultry (OR=0·61, *P*<0·05) meat consumption compared with non-parents. We did not identify significant relationships with race/ethnicity other than an increased frequency of African Americans reducing poultry consumption compared with Whites (OR=1·80, *P*<0·05), nor with education other than a reduced frequency of those with a bachelor’s degree or higher reducing seafood consumption compared with those with less than high-school education (OR=0·52, *P*<0·05). No relationships were seen with geography after controlling for other variables in the model and the latter results are not presented.

**Table 3:** Predictors of self-reported meat reduction in a nationally representative adult sample: USA, 2015

	Meat reducer		Red meat reducer		Processed meat reducer		Poultry reducer		Seafood reducer	
	OR	95 % CI	OR	95 % CI	OR	95 % CI	OR	95 % CI	OR	95 % CI
Age (*v*. 18–29 years)										
30–44 years	1·22	0·78, 1·89	1·30	0·82, 2·05	0·93	0·60, 1·44	0·99	0·58, 1·68	0·99	0·60, 1·63
45–59 years	2·04***	1·35, 3·10	1·58*	1·06, 2·35	1·67**	1·11, 2·51	0·99	0·61, 1·60	1·28	0·82, 1·99
≥60 years	1·67*	1·09, 2·56	1·42	0·95, 2·14	1·40	0·93, 2·12	1·06	0·65, 1·74	1·49	0·95, 2·35
Household income (*v*. ≥$US 75 000)										
≤$US 24 999	1·60*	1·00, 2·54	1·15	0·75, 1·78	0·88	0·58, 1·34	2·44***	1·52, 3·91	1·75**	1·12, 2·73
$US 25 000–49 999	1·39	0·95, 2·05	1·23	0·85, 1·78	1·10	0·76, 1·60	1·43	0·89, 2·28	1·35	0·90, 2·04
$US 50 000–74 999	1·03	0·71, 1·51	0·97	0·66, 1·41	0·90	0·62, 1·31	1·38	0·86, 2·22	1·54*	1·02, 2·33
Male	0·71*	0·53, 0·95	0·89	0·67, 1·17	0·90	0·68, 1·18	0·95	0·67, 1·34	0·95	0·70, 1·30
Parent (child aged 0–17 years in household)	0·68*	0·48, 0·98	0·63**	0·43, 0·91	0·90	0·63, 1·29	0·61*	0·38, 0·97	0·82	0·55, 1·23
Race/Ethnicity (*v*. White, Non-Hispanic)										
African-American, Non-Hispanic	1·00	0·61, 1·64	1·24	0·78, 1·98	0·95	0·59, 1·52	1·80*	1·08, 2·99	1·34	0·79, 2·25
Hispanic	1·24	0·78, 1·98	1·55	0·98, 2·45	1·47	0·94, 2·30	1·61	0·95, 2·73	1·53	0·97, 2·42
Other, ≥2 races	0·84	0·47, 1·49	1·04	0·60, 1·82	0·98	0·54, 1·77	1·64	0·88, 3·05	1·44	0·80, 2·58
Education (*v*. less than high school)										
High school	0·85	0·50, 1·45	1·11	0·64, 1·92	0·77	0·46, 1·29	0·69	0·38, 1·27	0·84	0·49, 1·44
Some college	1·04	0·60, 1·81	1·31	0·75, 2·29	0·92	0·54, 1·56	0·85	0·45, 1·61	0·86	0·49, 1·49
Bachelor’s degree or higher	1·19	0·69, 2·07	1·50	0·85, 2·65	1·17	0·69, 2·01	0·86	0·45, 1·62	0·52*	0·3, 0·93
Frequency, red meat	0·72***	0·62, 0·83	0·59***	0·51, 0·68	0·82***	0·71, 0·94	0·67***	0·56, 0·79	0·79***	0·68, 0·92
Frequency, processed meat	0·89	0·77, 1·03	0·99	0·86, 1·15	0·65***	0·56, 0·75	0·99	0·82, 1·19	0·92	0·79, 1·08
Often eats cheese/dairy in meatless meals	0·73***	0·61, 0·88	0·75***	0·63, 0·89	0·67***	0·56, 0·80	0·79*	0·66, 0·96	1·04	0·88, 1·23
Often eats vegetables in meatless meals	1·28***	1·09, 1·51	1·32***	1·13, 1·55	1·39***	1·18, 1·63	1·05	0·88, 1·26	0·89	0·75, 1·05

Results from multivariable logistic regressions. *P<0·05, **P<0·01, ***P<0·001.

Several meat consumption variables were examined as predictors of meat reduction. More frequent consumers of red meat were less likely to report reduced consumption of every type of meat (overall: OR=0·72, *P*<0·001; red: OR=0·59, *P*<0·001; processed: OR=0·82, *P*<0·001; poultry: OR=0·67, *P*<0·001; seafood: OR=0·79, *P*<0·001). More frequent consumers of processed meat, however, were only less likely to report reduced processed meat consumption (OR=0·65, *P*<0·001). Those whose meatless meals often included cheese or dairy were relatively unlikely to reduce consumption of overall (OR=0·73, *P<*0·001), red (OR=0·75, *P<*0·001), processed (OR=0·67, *P<*0·001) and poultry (OR=0·79, *P<*0·001) meat. Respondents replacing meat with vegetables were more likely to reduce overall meat consumption (OR=1·28, *P*<0·001) and consumption of red (OR=1·32, *P*<0·001) and processed meats (OR=1·39, *P*<0·001). No other foods were significantly associated with meat reduction.

### Reasons for meat reduction choices

**Fig. 1:**
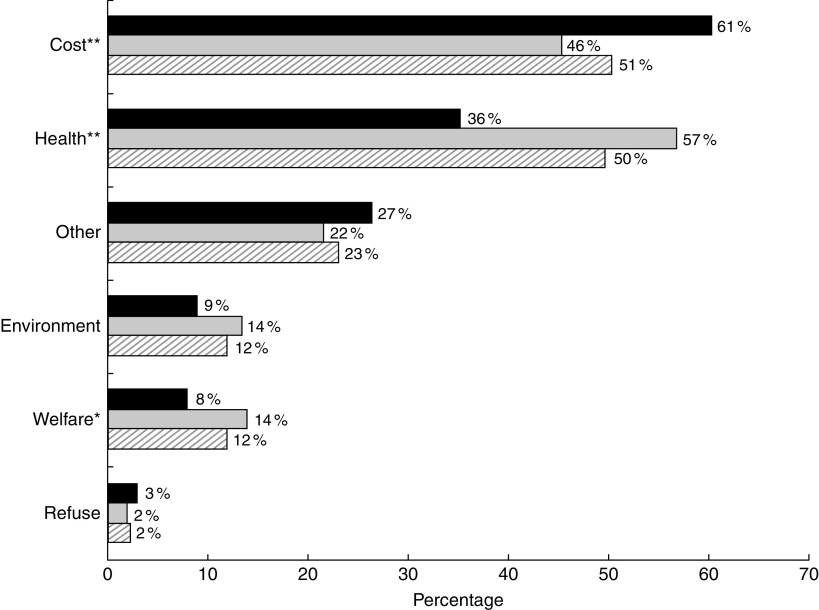
Reasons for meat reduction by income (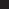, <$US 40 000; 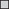, ≥$US 40 000; 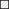, overall) in a nationally representative adult sample: USA, 2015. Figure depicts the stratified results for percentage indicating each item was a reason for meat reduction, by income. *P* values reflect χ^2^ for cross-tabulation: *P<0·05, **P<0·01, ***P<0·001

Meat reducers were asked why they reduced consumption, with the option to select multiple reasons (Fig. 1). Supporting our hypothesis, about half of participants indicated cost and health were reasons (51 and 50 %, respectively). Cost was substantially more important for those with lower income than higher (61 *v*. 46 % stratified results, *P*<0·01 for cross-tabulation), while health was substantially more important for those with higher incomes than lower (57 *v*. 36 %, *P*<0·001). Twelve per cent each said they reduced their meat consumption out of concern for animal welfare or the environment. Concerns about environment and cost were linearly associated with reduced red and processed meat consumption (*P*<0·01 and *P*<0·001, respectively).

**Fig. 2:**
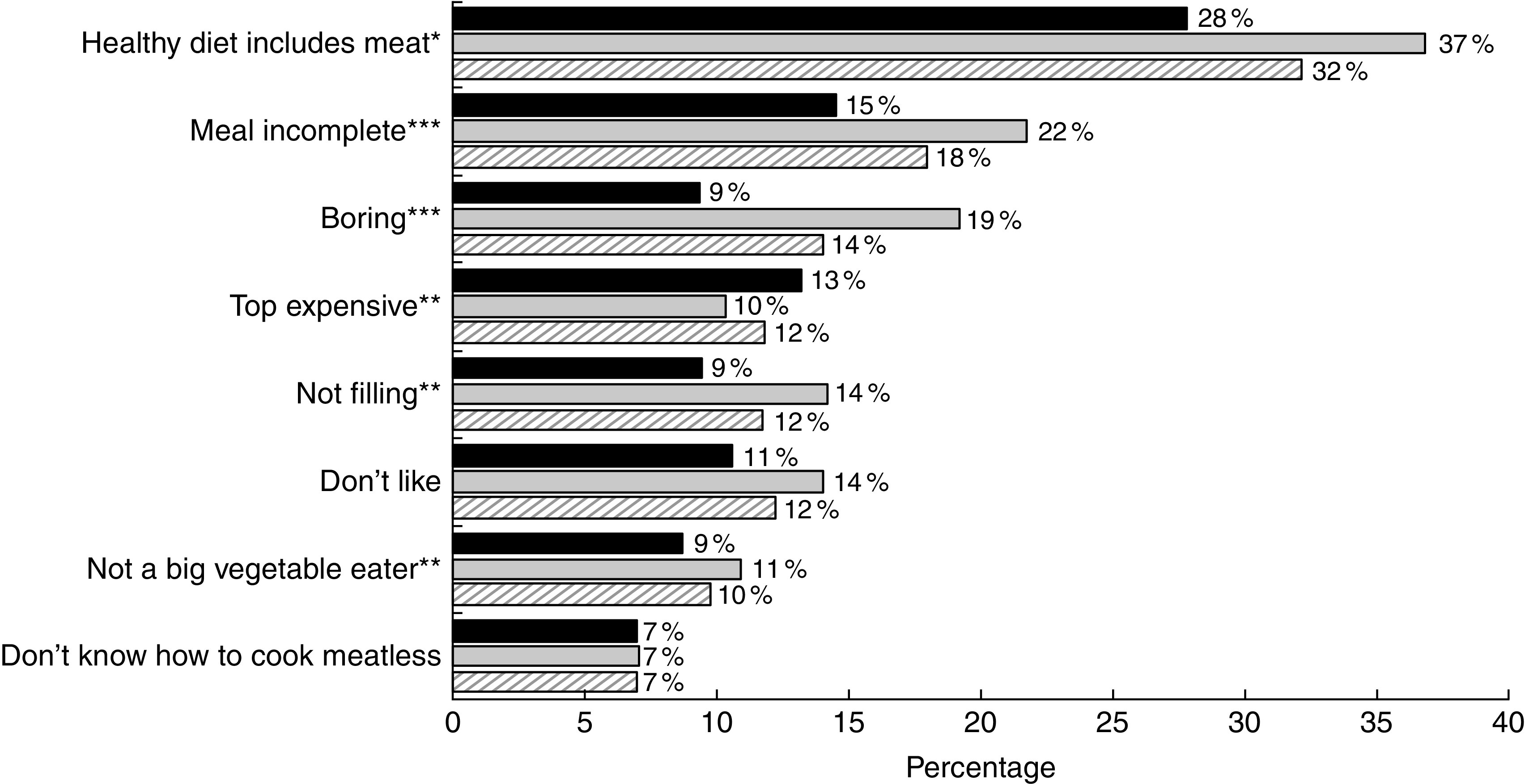
Non-meat reducers’ agreement with statements reflecting possible reasons for non-reduction by gender (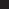, female; 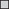, male; 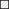, total) in a nationally representative adult sample: USA, 2015. Figure depicts stratified results for percentage who ‘agree’ plus ‘strongly agree’ by gender. *P* values reflect cross-tabulation comparison across all response options: *P<0·05, **P<0·01, ***P<0·001

Figure 2 shows non-meat reducers’ agreement with possible reasons for not reducing meat consumption, overall and stratified by gender. Perceptions that a healthy diet includes meat (32 %) or that meatless meals are incomplete (18 %) were the most common. Only 7 % agreed that not knowing how to cook was a reason they had not reduced their meat consumption. This sentiment was more frequent among non-parents than parents (5·6 *v*. 1·4 %, *P*<0·001). Men were more likely than women to say that they considered meat part of a healthy diet (37 *v*. 28 % stratified, *P*<0·05 for cross-tabulation), that meals were incomplete (22 *v*. 15 %, *P*<0·001), boring (19 *v*. 9 %, *P*<0·001) or not filling without meat (14 *v*. 9 %, *P*<0·01), and that they were not big vegetable eaters (11 *v*. 9 %, *P*<0·001). Men were less likely to say they considered meatless meals too expensive (10 *v*. 13 %, *P*<0·01). No significant differences were found by income.

### Low- and no-meat meals

**Fig. 3:**
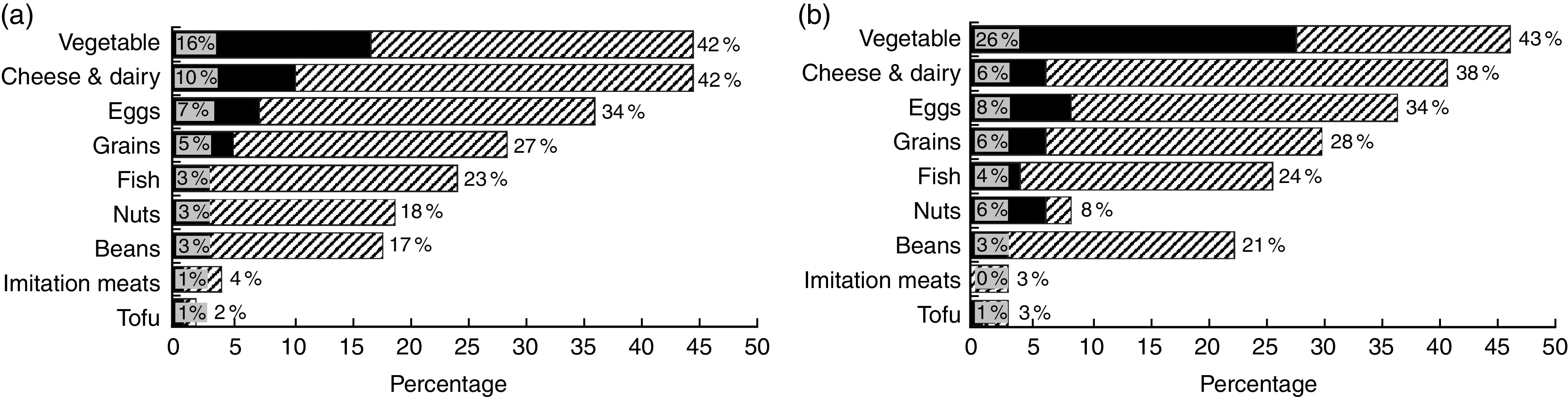
Reported frequency of foods eaten in non-meat meals, for (a) non-meat reducers and (b) meat reducers (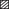, often; 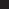, always), in a nationally representative adult sample: USA, 2015. Figure depicts stratified percentage of ‘often’ and ‘always’ responses to the question ‘For meals without meat, how often do you eat _____’, by meat reduction behaviour

Figure 3 describes what respondents reported eating in their meatless meals. Results suggest that individuals varied their meatless meals. Vegetables were most likely to be eaten frequently (65 %), followed by cheese/dairy (46 %) and eggs (41 %). Tofu and imitation meats were consumed the least frequently (4 %). Meat reducers were especially likely to eat vegetables (47 *v.* 19 %, *P*<0·01), while non-reducers were more likely to eat cheese and dairy (52 *v*. 44 %, *P*<0·01).

**Fig. 4:**
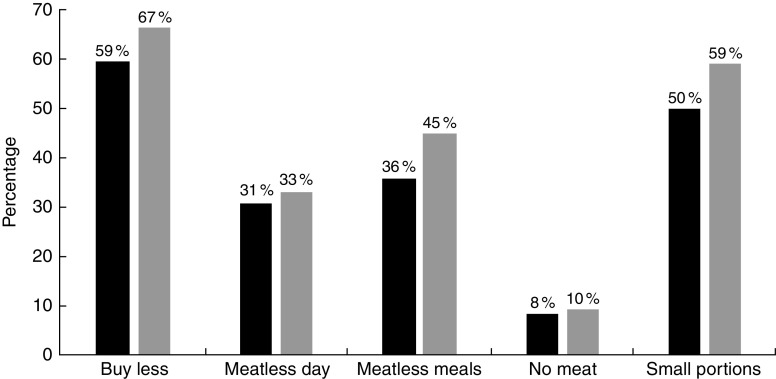
Approaches to reducing meat consumption (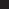, <$US 40 000; 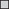, ≥$US 40 000) in a nationally representative adult sample: USA, 2015. Figure depicts the stratified results for percentage indicating each item was an approach to reducing meat reduction, by income

We also asked meat reducers about their approaches to reduction (Fig. 4). The most common approach was buying less meat (64 %) followed by smaller portion sizes (56 %), meatless meals (42 %), meatless days (32 %) and eschewing meat altogether (9 %).

## Discussion

The present survey described attitudes and approaches towards meat reduction in a nationally representative US adult sample. Two-thirds of respondents reported reducing consumption of at least one meat type across three years. Concerns about health and costs were primary motivations for reduction, while a primary reported reason for consuming meat was a belief that it is necessary for a healthy diet. These findings could inform targeted public health communication campaigns about reducing meat consumption and promoting affordable and healthy alternatives.

The present study provides the first US data about what people say they eat in non-meat meals. Beneficially for health and environment, nearly one-quarter reported ‘always’ eating vegetables in non-meat meals and another 42 % said they ‘often’ did. The next most common food, however, was cheese and dairy, which is often less healthful and less environmentally friendly. Additionally, meat reducers indicated more frequently eating beans, grains, fish and vegetables than non-reducers, although only vegetables were statistically significant. By contrast, non-reducers were especially likely to eat cheese/dairy in non-meat meals. These findings differ from those in a European study, which found more frequent reporting of cheese and much less of eggs, imitation meats, beans and tofu (that study did not include vegetables) in non-meat meals than we did^(^ ^23^ ^)^. Our findings are generally consistent, however, with a NHANES analysis finding higher Healthy Eating Index scores among those who did not eat meat on the two dietary recall dates *v.* those who did^(^ ^24^ ^)^.

Our findings of relatively infrequent consumption of beans and nuts, tofu and imitation meats suggest opportunities for building interest in these foods, which can be healthy alternatives providing fibre, Zn and Fe^(^ ^28^ ^–^ ^30^ ^)^. That said, recommendations to eat imitation meats such as sausages or burgers, however, should be tempered because these products can be high in Na, artificial flavourings, colourings, gums, sugar and preservatives^(^ ^31^ ^)^, although they can help make reduced-meat diets more appealing to some.

The reported frequent reductions in red and processed meat compared with other meats may result from successful public health messaging. More frequent meat reduction among women than men may reflect differential receptivity to these messages, possibly linked with the cultural association of meat with masculinity^(^ ^32^ ^)^. Lower meat reduction among parents may be related to the widely held view we identified among non-meat reducers, that meat belongs in a healthy diet.

Our findings regarding reasons for meat reduction are consistent with studies highlighting the resonance of cost and health, and indicating that the environment and animal welfare are seen as less important, although a UK study did find much more frequent concern for welfare than we did^(^ ^16^ ^,^ ^19^ ^,^ ^33^ ^,^ ^34^ ^)^. Low awareness and misconceptions regarding the environmental impacts of meat may partly explain the environment finding^(^ ^34^ ^)^.

Each of the four motivations for meat reduction (cost, health, environment, welfare) has limitations in terms of its potential to impact consumer decision making over time. For example, consumers motivated mainly by cost may stop reducing meat when prices decline or when the economy is robust^(^ ^35^ ^)^. We found that those with lower incomes were especially likely to reduce all types of meat, but they made greater reductions in poultry and seafood than red and processed meat. Accordingly, cost sensitivity may not drive shifts in the most important directions for health and environment.

Health motivations may also be unstable, as regular media consumers will be exposed to contradictory messages about risks and benefits of meat consumption. Evidence suggests ‘benefit’ messages often reduce the credibility of ‘risk’ messages^(^ ^36^ ^)^. Lastly, consumers with environmental or welfare concerns may switch to ‘better meats’ promoted as sustainable or animal-friendly. These products may still have negative effects, because marketing can be misleading and because environmental impacts are often mixed. For example, despite the many environmental benefits of grass-fed beef, the greenhouse gas emissions differ little from feedlot beef^(^ ^37^ ^)^. It is unknown whether purchasers of ‘better meats’ reduce overall meat consumption due to the cost. Consumers with higher incomes may be most willing to pay for these products^(^ ^38^ ^)^ and also be relatively likely to afford them frequently.

Given our findings, meat reduction campaigns should advise about preferred foods to eat when reducing meat and should address several common beliefs among non-meat reducers. They should highlight that diets lower in meat and higher in plant-based foods tend to have lower saturated fat and energy, and more fibre and other vitamins and minerals. Such diets may have less protein, but average US protein intake is higher than required^(^ ^39^ ^)^. The Academy of Nutrition and Dietetics states that ‘appropriately planned vegetarian, including vegan, diets are healthful, nutritionally adequate, and may provide health benefits for the prevention and treatment of certain diseases … for all stages of the life cycle’^(^ ^40^ ^)^. Campaigns should also educate about meat production environmental impacts. They should emphasize that meatless meals can be interesting and taste good, and should provide recipes.

The study strengths include the rigorous nationally representative sampling and detailed questions that provide the richest profile available to date regarding attitudes towards meat among reducers and non-reducers in the USA. To our knowledge, the survey is the first to gather data on what US meat reducers eat in their non-meat meals. The study also has several limitations. The questionnaire did not allow assessing overall meat consumption frequency, but rather only the frequency of consuming each meat type. The resultant low frequency by item could be misleading. The instrument also did not identify vegetarians and vegans.

Meat reduction frequencies reported in the present survey and others contrast with US Department of Agriculture data indicating stable meat production levels from 2011 to 2013^(^ ^39^ ^)^. We note however that production and consumption are not directly connected, due to trade and other factors. Also, our definition of meat reducer may account for some of the discrepancy because it included the possibility of, for example, reducing red meat while increasing poultry. Another possibility is increased consumption by non-reducers, and counterbalancing reduction in other meals or via portion size. Lastly, all survey findings are subject to biases in self-report and recall, sampling, non-response, question wording and response option structure, and errors in post-survey weight construction. That said, the survey research firm, GfK, makes strong efforts to assure national representativeness.

## Conclusions

Our findings shed light on meat-related population behaviours and attitudes towards meat reduction. We find that Americans report reducing their meat consumption compared with three years ago, with reductions of red and processed meat being most frequent. However, persistent perceptions, such as regarding meat as necessary for a healthy diet, may challenge efforts to further reduce meat consumption in the name of both population and environmental health. Public health messaging to encourage meat reduction should emphasize the health and environmental benefits of plant-based diets and make concrete suggestions for meat substitutes.

Understanding how the public – in the USA and other high meat-consuming countries globally – approaches these dietary choices is essential for advancing communication campaigns to encourage reduced meat consumption and replacement with healthy and environmentally friendly alternatives.

## Supplementary material

For supplementary material accompanying this paper visit https://doi.org/10.1017/S1368980017004190. click here to view supplementary material
