# Supplementary material for: Reducing meat consumption in the USA: a nationally representative survey of attitudes and behaviours
Source: Public Health Nutr. 2018 Mar 26;21(10):1835–44. doi: 10.1017/S1368980017004190 (PMC6088533; doi:10.1017/S1368980017004190)
Supplement: Supplementary file 1 [file S1368980017004190sup.zip › S1368980017004190sup001.docx]

**Supplemental File 1: Relevant Questions from Survey**

In the past 7 days, how often did you eat…?

| Not at all | One time | 2 to 4 times | 5 to 6 times | Once a day | 1 to 2 times a day | 2 to 3 times a day | At every meal |
| --- | --- | --- | --- | --- | --- | --- | --- |
| 1 | 2 | 3 | 4 | 5 | 6 | 7 | 8 |

1. Fresh or frozen red meat (beef, pork, lamb, duck, etc.)

2. Processed meat (bacon, hot dogs, deli meats, sausages, etc.)

3. Fresh, frozen or canned/bagged poultry (chicken, turkey, etc.)

4. Fresh, frozen or canned/bagged seafood (fish, shrimp, crab, clams, etc.)

Compared to three years ago, the amount of _________ I eat now is …

| A lot less | Slightly less | About the same | Slightly more | A lot more |
| --- | --- | --- | --- | --- |
| 1 | 2 | 3 | 4 | 5 |

1. Fresh or frozen red meat (beef, pork, lamb, duck, etc.)

2. Processed meat (bacon, hot dogs, deli meats, sausages, etc.)

3. Fresh, frozen or canned/bagged poultry (chicken, turkey, etc.)

4. Fresh, frozen or canned/bagged seafood (fish, shrimp, crab, clams, etc.)

Did you eat less meat by _____?

| Yes | No |
| --- | --- |
| 1 | 2 |

A. Eating smaller portions of meat?

B. Buying less meat?

C. Eliminating meat from a specific meal or meals?

D. Cutting meat out of your diet one day each week?

E. Cutting meat out of your diet entirely?

For meals that don’t have meat, how often do you eat ___________?

| I don’t eat | Rarely | Sometimes | Often | Always |
| --- | --- | --- | --- | --- |
| 1 | 2 | 3 | 4 | 5 |

1. Fake meats (such as meat-free nuggets, crumbles or strips, veggie burgers or vegetarian sausages)

2. Cheese or other dairy

3. Eggs

4. Nuts

5. Fish or seafood

6. Tofu, seitan or tempeh

7. Lentils or beans

8. Grains (such as rice, quinoa, or barley)

9. Vegetables

What, if any, of the following reasons help to explain the change in the amount of meat you eat?

1 Health effects of meat consumption

2 Animal welfare issues involved with meat products

3 Environmental impacts of meat production

4 The cost of meat

5 Some other reason

When you think about eating less meat, how strongly do you agree or disagree with the following statements?

| Strongly disagree | Disagree | Somewhat disagree | Neither agree or disagree | Somewhat agree | Agree | Strongly agree |
| --- | --- | --- | --- | --- | --- | --- |
| 1 | 2 | 3 | 4 | 5 | 6 | 7 |

1. I don’t know how to cook meatless meals.

2. I / my family don’t like how meatless meals taste.

3. It is too expensive.

4. I believe a healthy diet includes meat.

5. Meatless meals are boring.

6. I am not a big vegetable eater.

7. A meal is not complete without meat.

8. Meatless meals are not filling.
