# Supplementary material for: Reducing meat consumption in the USA: a nationally representative survey of attitudes and behaviours
Source: Public Health Nutr. 2018 Mar 26;21(10):1835–44. doi: 10.1017/S1368980017004190 (PMC6088533; doi:10.1017/S1368980017004190)
Supplement: Supplementary file 1 [file S1368980017004190sup.zip › S1368980017004190sup002.docx]

**Supplemental File 2: Checklist for Reporting Results of Internet E-Surveys (CHERRIES)**

Citation: Eysenbach, G. Improving the Quality of Web Surveys: The Checklist for Reporting Results of Internet E-Surveys (CHERRIES). *Journal of Medical Internet Research*. 2004;6(3):e34. doi:10.2196/jmir.6.3.e34.

*NOTE: All included quotes are drawn from GFK Knowledgeworks’ “Documentation for Human Subject Review Committees,” 2013.* [*http://www.knowledgenetworks.com/ganp/irbsupport/*](http://www.knowledgenetworks.com/ganp/irbsupport/)

| 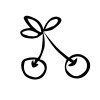 | **Checklist for Reporting Results of Internet E-Surveys (CHERRIES)** | |
| --- | --- | --- |
| ***Item Category*** | ***Checklist Item*** | ***Explanation*** |
| **Design** |  |  |
|  | Describe survey design | Target population: US adult population (over age 18), 2014.  Sampling frame: US households. GfK maintains an online panel of approximately 50,000 members recruited using equal-probability address-based sampling. The sampling frame covers 97% of US households [including those with unlisted telephone numbers or without landlines]. To improve representation, GfK provides Internet access and devices to those who lack them, and oversamples census blocks with high percentages of African American and Hispanic residents. In addition to pre-survey weighting to reflect annually updated benchmark US population distributions, GfK provides post-survey sample weights based on seven variables to correct for sampling and non-response biases.  Approximately 60% of panel households recruited through post-2009 address-based sampling (ABS) frame and 40% through pre-2009 random-digit dial (RDD) methodology.  “**ABS Recruitment.** ABS involves probability-based sampling of addresses from the U.S. Postal Service’s Delivery Sequence File. The key advantage of the ABS sample frame is that it allows sampling of almost all U.S. households. An estimated 97% of households are “covered” in sampling nomenclature. Regardless of household telephone status, those households can be reached and contacted through postal mail. In late 2009 the ABS sample began incorporating a geographic stratification design. Census blocks with high density minority communities were oversampled (Stratum 1), and the balance of the census blocks (Stratum 2) were relatively under-sampled. The definition of high density and minority community and the relative proportion between strata differed among specific ABS samples. In 2010, the two strata were redefined to target high density Hispanic areas in Stratum 1 and all else in Stratum 2. In 2011, pre-identified ancillary information, rather than census block data, were used to construct and target four strata as follows: Hispanic ages 18-24, non-Hispanic ages 18-24, Hispanic ages 25+, and non-Hispanic ages 25+. Also in 2011, a separate sample targeting only persons ages 18-24 was fielded across the year, again using predictive ancillary information. Combined with the four-stratum sample, the base weight adjustment compensates for cases from this unique young adult oversample. In 2012, a similar four-stratum design was implemented, with the ages changed to 18-29 and 30+ for both the Hispanic and non-Hispanic strata. For every survey sample, an appropriate base weight adjustment is applied to each relevant sample to correct for these stratified designs.”  **“RDD Recruitment.** For panel members who were recruited using RDD-based sampling (pre-April 2009), list-assisted RDD sampling techniques were used on the sample frame consisting of the entire U.S. residential telephone population. Only banks of telephone numbers (each consisting of 100 telephone numbers) that had zero or one directory-listed phone numbers were excluded. Two strata were defined using 2000 Census Decennial Census data which were appended to all telephone exchanges. The first stratum had a higher concentration of Black and Hispanic households, and the second stratum had a lower concentration of these groups relative to the national estimates. Telephone numbers were selected with equal probability of selection for each number within each of the two strata, with the higher concentration Black and Hispanic stratum being sampled at approximately twice the rate of the other stratum. The sampling was done without replacement to ensure that numbers already fielded would not be fielded again.” |
| **IRB (Institutional Review Board) approval and informed consent process** |  |  |
|  | IRB approval | This study was approved by the [name removed for blind review] School of Public Health Institutional Review Board  . |
|  | Informed consent | GfK states, “On rare occasions, Human Subjects Review Committees have required that GfK obtain informed consent for a specific survey, most typically those dealing with highly sensitive topics.” In our case, the IRB did not request such consent due to the low-risk subject matter. The IRB also did not request that specific information about the investigator be provided to subjects. The first content displayed in the survey was, “In this survey, we will ask you questions about cooking and food. The survey should take less than 15 minutes to complete. We ask you to please try to take this survey in one sitting.”  In signing up for the GfK panel, participants are told, “Your email address will be protected by our privacy standards. We can promise you that GfK will never share your email address with anyone without your permission.” |
|  | Data protection | No personal information was collected or stored. |
| **Development and pre-testing** |  |  |
|  | Development and testing | The survey was developed using both new questions and questions adapted from sources including cited previous polling surveys. The instrument was reviewed by content experts and pilot tested (including usability and technical functionality of the electronic questionnaire) using a random sample of 25 participants in the survey firm’s panel. |
| **Recruitment process and description of the sample having access to the questionnaire** |  |  |
|  | Open survey versus closed survey | Closed survey |
|  | Contact mode | Contact was made with panel members by email. |
|  | Advertising the survey | The survey was only available to the members of the panel that were selected based on GfK Knowledgeworks’ algorithm. |
| **Survey administration** |  |  |
|  | Web/E-mail | The survey was web-based. |
|  | Context | As noted, the survey was not advertised, and was only available to selected panel members. |
|  | Mandatory/voluntary | The survey was voluntary. |
|  | Incentives | “**incentives** are used to maintain a high degree of panel loyalty and to prevent attrition from the panel. The ‘panel loyalty’ incentive is the web-enabled devices and the Internet connections that GfK provides for free to households without these items. Panelists who use their own personal computers and Internet service for survey participation are enrolled in a points program that is analogous to a ‘frequent flyer’ program, in that respondents are credited with points in proportion to their regular participation in surveys. Panelists can redeem their points at times of their own choosing, with general accumulations of $4 to $6 per month.” |
|  | Time/Date | Pre-test: March 20-23, 2015; Main survey: March 31-April 27, 2015. |
|  | Randomization of items or questionnaires | Some items were randomized in order to prevent bias, where appropriate, as shown in the survey instrument (Supplement 1). |
|  | Adaptive questioning | Adaptive questioning was used. |
|  | Number of Items | The number of questions per page was determined by GfK’s staff, who are experienced in survey design and administration, as demonstrated by their multiple rounds of feedback. |
|  | Number of screens (pages) | As above, the number of screens was determined by GfK’s experienced staff. We do not have this number in our records, but GfK may maintain it. |
|  | Completeness check | We do not have information in our records regarding whether the firm does completeness checks. |
|  | Review step | We do not have information in our records regarding whether respondents were able to review and change their answers. |
| **Response rates** |  |  |
|  | Unique site visitor | NA because respondents were part of panel. |
|  | View rate (Ratio of unique survey visitors/unique site visitors) | NA because respondents were part of panel. |
|  | Participation rate (Ratio of unique visitors who agreed to participate/unique first survey page visitors) | 1,568 were sampled for the main survey and 1,137 completed the main survey for a completion rate of 73% |
|  | Completion rate (Ratio of users who finished the survey/users who agreed to participate) | 1,112 were determined to be valid responses. The 25 excluded cases had completed the survey in under 4 minutes. |
| **Preventing multiple entries from the same individual** |  |  |
|  | Cookies used | NA because respondents were part of panel and would not receive repeat invitations. |
|  | IP check | NA because respondents were part of panel and would not receive repeat invitations. |
|  | Log file analysis | NA because respondents were part of panel and would not receive repeat invitations. |
|  | Registration | NA because respondents were part of panel and would not receive repeat invitations. |
| **Analysis** |  |  |
|  | Handling of incomplete questionnaires | "Not asked" responses are recoded as "-2" and "Refused" cases are coded as "-1".” |
|  | Questionnaires submitted with an atypical timestamp | Surveys completed in under 4 minutes were excluded based on GfK Knowledgeworks’ standard practice. |
|  | Statistical correction | In addition to pre-survey weighting to reflect annually updated benchmark US population distributions, GfK provides post-survey sample weights based on seven variables to correct for sampling and non-response biases. |
